# Identification and analysis of novel salt responsive candidate gene based SSRs (cgSSRs) from rice (*Oryza sativa* L.)

**DOI:** 10.1186/s12870-015-0498-1

**Published:** 2015-05-16

**Authors:** Kutubuddin Ali Molla, Ananda Bhusan Debnath, Showkat Ahmad Ganie, Tapan Kumar Mondal

**Affiliations:** Division of Genomic Resources, National Bureau of Plant Genetic Resources, IARI Campus, Pusa, New Delhi 110012 India

**Keywords:** Microsatellite, Genic-SSR, cgSSR, Salt tolerance, Salt responsive gene, Rice, Molecular diversity, Candidate gene, Rice genotype

## Abstract

**Background:**

Majority of the Asian people depend on rice for nutritional energy. Rice cultivation and yield are severely affected by soil salinity stress worldwide. Marker assisted breeding is a rapid and efficient way to develop improved variety for salinity stress tolerance. Genomic microsatellite markers are an elite group of markers, but there is possible uncertainty of linkage with the important genes. In contrast, there are better possibilities of linkage detection with important genes if SSRs are developed from candidate genes. To the best of our knowledge, there is no such report on SSR markers development from candidate gene sequences in rice. So the present study was aimed to identify and analyse SSRs from salt responsive candidate genes of rice.

**Results:**

In the present study, based on the comprehensive literature survey, we selected 220 different salt responsive genes of rice. Out of them, 106 genes were found to contain 180 microsatellite loci with, tri-nucleotide motifs (56%) being most abundant, followed by di-(41%) and tetra nucleotide (2.8%) motifs. Maximum loci were found in the coding sequences (37.2%), followed by in 5′UTR (26%), intron (21.6%) and 3′UTR (15%). For validation, 19 primer sets were evaluated to detect polymorphism in diversity analysis among the two panels consisting of 17 salt tolerant and 17 susceptible rice genotypes. Except one, all primer sets exhibited polymorphic nature with an average of 21.8 alleles/primer and with a mean PIC value of 0.28. Calculated genetic similarity among genotypes was ranged from 19%-89%. The generated dendrogram showed 3 clusters of which one contained entire 17 susceptible genotypes and another two clusters contained all tolerant genotypes.

**Conclusion:**

The present study represents the potential of salt responsive candidate gene based SSR (cgSSR) markers to be utilized as novel and remarkable candidate for diversity analysis among rice genotypes differing in salinity response.

**Electronic supplementary material:**

The online version of this article (doi:10.1186/s12870-015-0498-1) contains supplementary material, which is available to authorized users.

## Background

Rice (*Oryza sativa* L.) is the most widely consumed staple food by over half of the world’s population and it provides 27 percent of dietary energy supply worldwide [1]. The burgeoning world population growth and shrinkage of agricultural land are the two main reasons of an estimated food shortage in the coming days. Rice production must increase at least 25% by 2030 in order to feed the estimated world population [2]. The situation is more aggravated due to the huge loss of crop yield as a result of different abiotic stresses. Soil salinity, one of the top most abiotic stresses, imposes limitation to the growth and development of rice plant causing yield losses of more than 50 percent [3]. Rice being a natural glycophyte, for every unit of excess salinity (deciSiemens/metre), rice yields are estimated to reduce over 12 percent [4]. In contrast to animal, plants, the creature of nature, are unable to move from one place to other compelling them to endure the stress in standing condition. In this scenario, rice genetic improvement is one of the top priority areas to increase yield overcoming those constraints to meet the future demand.

Marker assisted selection remarkably speeds up the efficiency and preciseness of breeding programme over the traditional breeding. Availability of high quality genome sequence [5] further eases up the mining of DNA markers to facilitate marker assisted breeding programme in rice. With the advancement of molecular techniques, a diverse group of molecular markers like restriction fragment length polymorphism (RFLP), random amplification of polymorphic DNA (RAPD), variable number tandem repeat (VNTR), amplified fragment length polymorphisms (AFLP), microsatellites polymorphism or simple sequence repeats (SSR) and single nucleotide polymorphism (SNP) have been developed. Among all, SSR markers are outstanding in application because of their high reproducibility, multi-allelic nature, codominant inheritance, good uniform genome coverage, simplicity and inexpensive developmental methodology [6]. SSRs are present in the genome as tandem arrays of short nucleotide repeats usually 1–5 bases per unit. SSR markers have been extensively used in phylogenetic relationship cum diversity analysis among rice genotypes [7-9], association mapping [10,11] and identification and characterization of important trait related QTL [12-14].

Traditional SSR markers developed from random genomic sequences have uncertainty of linkage with the transcribed regions (genes) of the genome, whereas genic SSR derived from expressed sequence tag (EST) or candidate gene sequences based SSR have far better possibility of linkage to important loci conferring desired phenotypes [15]. Genic SSR markers are highly valuable by virtue of their high transferability to related species, usefulness in functional diversity analysis and utilization as anchor markers for comparative mapping and evolutionary studies [16]. Genic SSR markers were developed from EST sequences available from public database in different crop species like rice [17,18], wheat [19], barley [20], date palm [21], common bean [22] and many others. As another approach, development of SSR markers based on important candidate genes related to a particular trait may greatly expedite marker assisted breeding programme for the trait. Moreover, looking for SSR in candidate genes may attain many unanswered question about the regulation of those genes as increasing evidences are being reported about the regulatory roles of microsatellites in gene sequences [23-25]. However, report on the development of genic SSR marker based on candidate gene sequences (cgSSR) are scanty [26,27].

From literature survey, around 220 different genes in rice were found to be salt responsive as evidenced by forward/reverse genetics study. To the best of our knowledge, there is no report on the development of candidate gene based SSR markers in rice. In this study, we report an exclusive identification of novel salt responsive candidate gene based SSR markers (cgSSRs) from rice. We extensively investigated all characterized salt responsive rice genes from published reports. When those gene sequences were subjected for mining SSR, 106 genes were found to contain simple sequence repeats. Among those cgSSRs, 19 primer sets were evaluated and validated for their extent of polymorphism in 17 salt tolerant and 17 salt sensitive rice genotypes. The originated dendrogram revealed their remarkable ability to distinguish rice genotype on the basis of salinity response. This is the first report of salt responsive candidate gene based SSR (cgSSR) marker identification and validation in rice.

## Methods

### Plant materials

A total of 34 rice genotypes including two contrasting panel (salt tolerant and salt susceptible), each of which contains 17 genotypes, were subjected for the polymorphism survey in this study. Details of rice genotypes along with their salt sensitivity level are given in Additional file 1.

### Isolation of genomic DNA

Fresh green leaves were collected, weighed (100 mg) and immediately used for DNA isolation or stored at −80°C after snap freezing in liquid N_2_. DNA was isolated following a previously described protocol [28]. Leaf tissues were grinded to fine powder employing liquid N_2_ in a pre-chilled morter. Prewarmed CTAB buffer (2.0% CTAB (w/v); 0.1 M Tris Cl, pH 8; 0.02 M of EDTA, pH 8; 1.4 M NaCl) was added to the powder for extraction and the mixture was incubated at 60°C for 20 min. Supernatant was collected after centrifugation and a solution of Chloroform: Isoamyl alcohol (24:1) was mixed. After centrifugation, aqueous phase was collected, mixed with equal volume of isopropanol and incubated for 20 min at −20°C. Centrifugation was done to pellet down DNA. Pellet was washed with 70% (v/v) ethanol, air dried and dissolved in nuclease free water. The sample was treated with RNase enzyme at 37°C and subsequently purified by phenol-chloroform method [28]. Concentration and quality of purified DNA were checked in Nanodrop spectrophotometer (Thermo scientific, USA) employing 260/280 and 260/230 ratio and also by 1% (w/v) agarose gel electrophoresis.

### Salt tolerant genes, SSRs mining and Primer designing

An extensive search of literature was performed manually to identify the rice candidate genes conferring salt tolerant phenotype. All rice genes which have been reported elsewhere to confer stable salt tolerance in transgenic plants on homologous and heterologous over expression and which showed either enhanced or suppressed expression upon salt stress were considered in this study. The gene bank locus numbers were retrieved and subsequently sequences of all those genes were downloaded from the web (http://rice.plantbiology.msu.edu/) resources of Rice Genome Annotation Project [29]. The gene sequences were used to mine SSRs in SSR identification tool [30]. Respective references of those candidate genes which have been found to contain microsatellite repeats were given in Additional file 2: Table S2. We designed primers from the flanking sequences of the identified microsatellite repeat region. Primers were designed manually with the following parameters: primer length 20–25 bp, melting temperature 55–60°C, GC percentage- 45–60 and product size- 130–250 bp. Details of the primers, melting temperature and the anticipated amplification product length are given in Table 1.

**Table 1:** **Details of salt tolerance gene, respective genbank LOC number, motifs with repeat number and location in sequence, primers with Tm and molecular weight of expected band**

**Gene bank LOC No.**	**Gene**	**Forward (Tm)**	**Reverse (Tm)**	**Expected amplicon size**	**Number of alleles**	**PIC value**	**Function**	(Motif) repeat*	**Location of motif**
LOC_Os11g08210	OsNac5	ATGTGATTAGAGTCGCTTTCAGTTGG (56.9C)	CCAGCTTGTACTTGTGCCAGCC (58.4C)	238 bp	10	0.110	TF	(TAA)_18_	3′UTR
LOC_Os10g25010	OsCML8	GAGAATCAGAGCAAGAGTCTGAACCAGC (61C)	GTCAGCCGCTTCTTCCTCACCTG (61C)	209 bp	21	0.275	Signaling	(CGG)_9_	CDS
LOC_Os01g32120	OsCML11	CATGCAAGCCTGCGGAGACG (61.2C)	CGGTCGAAGGAGCGGAAGATCT (60.5C)	154 bp	42	0.221	Signaling	(CAG)_10_	CDS
LOC_Os02g17500	OsGMST1	AGGAACCAACAGAAGCAAAGGTG (56.3C)	GAGGTGATTTGATGCTGTGAGGC (57.4C)	194 bp	25	0.289	Sugar Transporter	(AG)_10_	5′UTR
LOC_Os07g06740.2	OsCPK17	TTGCCTTTTGATCTAGTGCATTGG (57.2C)	GTCTTCGTCCTTTACTAAATAGCACTCC (55.8C)	267 bp	21	0.353	Kinase	(CT)_9_/(CT)_11_	3′UTR
LOC_Os02g04630	OsCAX (D)	CTGTTTGGCAATCTGCCAGC (55.6C)	CGTCTCGGCAAAATGTTCCTC (56.1C)	139 bp	20	0.326	Ion transporter	(CT)_18_	Intronic
LOC_Os02g04630	OsCAX (T)	CTTTGGTTGGTTCAGGACGATG (55.9)	GAATTGGAAGCTGTTGGCTCATTC (57.9)	163 bp	18	0.169	“	(TTA)_26_	Intronic
LOC_Os07g38090	OsC3H50	GAGGAATTAGACCATTTAACTCGTCGC (58.7)	GAATCCGACCCAATCCAATCAAG (58.3)	214 bp	24	0.199	RNA processing	(TC)_9_	5′UTR
LOC_Os06g48590.1	OsMAPK4	GACATCTAAGTGCCGCGTGTTC (56.2)	TACATGCAGCGTCGAATCGAAG (57.6)	254 bp	17	0.358	Kinase	(CT)_12_	5′UTR
LOC_Os01g54600	OsWRKY13	CCATGCGTACATACACGTTCATGTG (57C)	GATGGGTGCAGCTTTCAATGATC (57.3C)	246 bp	20	0.370	TF	(AG)_16_/(GA)_9_	5′UTR
LOC_Os01g72530.1	OsCML31	GTTGATGGATCTGTAAATGCTTCATGG (58.8)	GGCACCATGGAGCACCAAAC (57.4)	167 bp	Not amplified	Not amplified	Signaling	(AT)_40_	3′UTR
LOC_Os01g45274.1	OsCA1	CCATCGAGTACGCCGTCTGC (57.9)	CTTCACCATGAATGTTACACACCCTAC (56.8)	281 bp	35	0.296	Chloroplast photosynthesis	(CT)_9_	Intronic
LOC_Os02g02840.1	OsRacB (D)	GCTCCTCCTTCAACCTTCTTCTTTC (57.1C)	GTGACGCACTTTATGAACCTGGAC (56.5C)	176 bp	30	0.318	Signaling, GTPase	(GA)_21_	5′UTR
LOC_Os02g02840.1	OsRacB (T)	CAAGACCTGCATGCTCATCTCC (56.1C)	CCAGATCAAGAACCATAATCCTAGCTC (56.9C)	202 bp	14	0.386	“	(TTC)_9_	Intronic
LOC_Os05g51670.1	OsUGE1	CACAACGCCAACAACCTCGAC (57.7C)	GCTTATCGAGATGGGAATGGTTG (56.5)	154 bp	11	0.087	Nucleotide sugar metabolism	(TC)_9_	Intronic
LOC_Os06g48590.1	OsMSRMK3	CACCTCCATTTCCCATTCCACC (58.9C)	CGAATCGAAGGCGGCAGCTATAG (60.9)	201 bp	27	0.340	Signaling, Kinase	(CT)_12_	5′UTR
LOC_Os02g35190.2	OsCLC-1	CAGAGAAGCCAAGCAAAGAAAGTCTC (58.1C)	CCGTGCTCTCGATGTCGTAGTTG (59.2)	179 bp	24	0.322	Ion trasport	(AGA)_11_	5′UTR
LOC_Os09g13570	OsbZIP71	CTCAGTAAGCTCCCTGTAGTTGTAGCC (57.3)	GTTCAGGTCATCTTCCGACCTGG (58.5)	259 bp	13	0.323	TF	(TA)_12_	5′UTR
LOC_Os03g02590	OsPEX11-1	GCTGCTCTCGACTTTCTTGTTCC (56.2)	ACTAGCCCTGCACAGACTGAAGAG (55.8)	276 bp	21	0.261	Peroxisomal biogenesis	(TG)_19_	Intronic

D- di-nucleotide and T- tri-nucleotide. *subscript denotes the number of repeats.

### PCR amplification and 6% polyacrylamide gel electrophoresis

PCR amplification was done from 34 genotypes with 19 pairs of SSR primers in a total volume of 25 μl using a C1000 Thermal Cycler (Bio Rad, USA). Each 25 μl volume of reaction mixture contained 50 ng of genomic DNA as template, 1X Taq polymerase buffer, 2 mM MgCl_2_, 0.2 mM dNTPs mix, 0.4 pM each of the forward and reverse primer, 1 U of Taq polymerase. The optimized condition was initial 5 minutes incubation at 97°C for complete denaturation, followed by 38 cycles consisting of 94°C for 1 min, 55°C- 60°C (vary with the primer pair) for 1 min, 72°C for 2 min and finally 72°C for 10 min. The experiments were repeated twice.

Resolving of all PCR products were performed in a vertical 6% non denaturing Polyacrylamide gel electrophoresis (PAGE) system at constant 140 V with 1X TAE (Tris acetate EDTA) buffer (pH-8.0). The gel was stained with ethidium bromide solution and visualized in gel documentation system (Protein Simple, USA).

### Allele scoring and sequencing

Molecular weights of the amplified bands were determined based on the relative migration of standard 100 bp DNA ladder (Thermo scientific, USA) in the gel. The molecular weight of each allele was determined using the Alpha View software (Protein Simple, USA). Presence or absence of a particular allele was denoted as 1 or 0 respectively and the data was plotted to generate a data matrix for further analysis. When an allele was found exclusively in one genotype, it was designated as unique allele. Alleles found in less than 5% of genotypes were designated as rare.

DNA was eluted from the bands of selected alleles and purified using QIAEX II Gel Extraction Kit (Qiagen, Germany). The purified DNA was sequenced. The obtained sequences were aligned with the original target sequence using NCBI blastn tool.

### Data analysis

Analysis of data was performed according to the method described in a previous report [31]. Polymorphism information content (PIC) value of each primer pairs was calculated according to the formula: PIC = 1- ∑ *pi*^2^, where *pi* is equal to the frequency of the *i*th allele of a particular locus [32]. DARwin v5 software was used to draw the phylogenetic relationship among the rice genotypes [33]. Euclidean distance matrix was computed for evaluation of genetic distances between genotypes and further utilized to construct a dendrogram using the neighbour joining method [34]. Bootstrapping data over a locus for 1000 replications of the original matrix (1/0 data matrix) was used to evaluate the significance of each node. Principal coordinate analysis (PCoA) was carried out in DARwin v5 for differentiating the genotypes.

## Result

### Frequency and distribution of salt responsive cgSSRs

**Figure 1:**
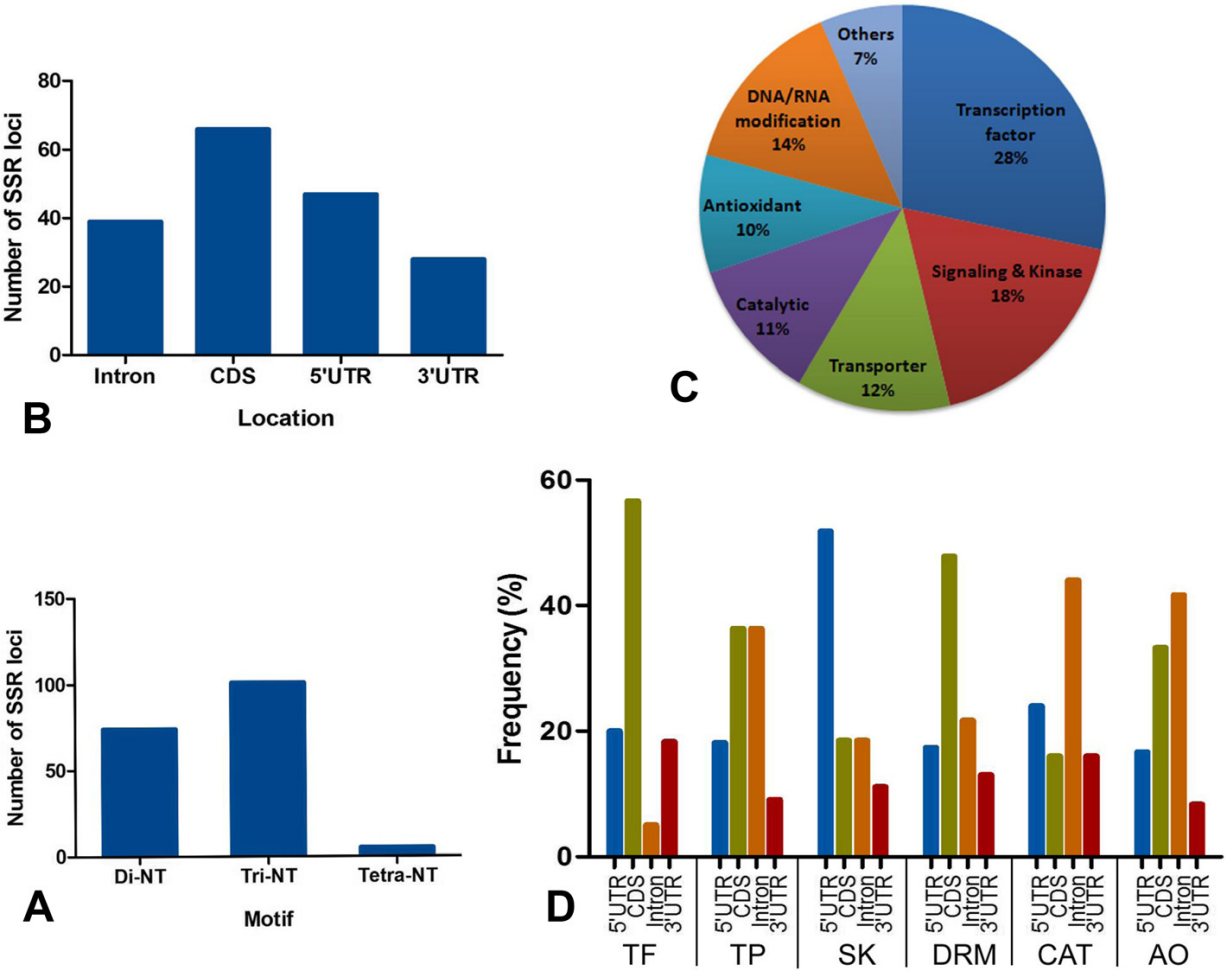
Frequency and distribution of salt responsive cgSSRs in rice. **A)** Number of different SSR motifs found, **B)** number of motifs found in different locations of salt responsive gene sequences, **C)** Percentage of different functional classes of salt responsive genes harbouring SSR loci. **D)** Location of SSR loci in each functional class of salt responsive genes. TF- transcription factor, TP- transporter, SK- signaling & kinase, DRM- DNA/RNA modifying, CAT- catalytic and AO- Antioxidant.

**Figure 2:**
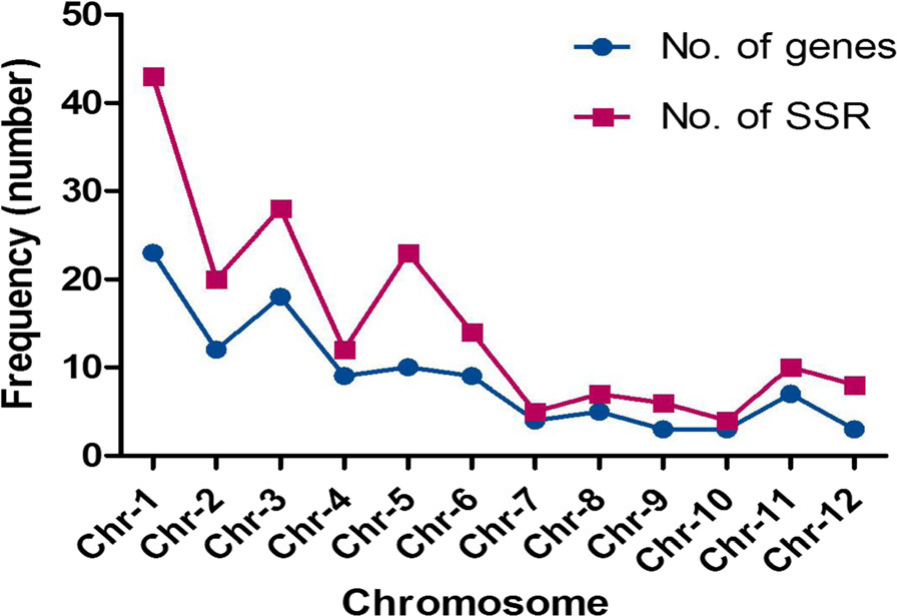
Frequency and distribution of salt responsive cgSSR loci in different rice chromosomes.

A total of 220 different salinity responsive candidate genes were screened for the presence of SSR which yielded a total of 180 SSR loci from 106 (48.18%) candidate genes. List of those genes harbouring SSR loci with their respective gene bank LOC number, function, number, types and location of motif found were detailed in Additional file 2. The study included only di-tetra nucleotide repeats and reiteration of motifs less than 5 times was excluded. Tri-nucleotide motifs were found to be the largest (56.11%) and tetra-nucleotide motifs formed the smallest group (2.8%) (Figure 1A). A total of 50 different kinds of motifs were found, of which, CT/TC motifs (12.8%) were most frequent, followed by AT/TA (10%) and CGG (7.7%) motif (Additional file 3). Among the trinucleotide repeat motifs, CGG (coding for arginine) and GCC (coding for alanine) were more abundant than others (Additional file 3). The number of repetition of a motif varied from 5–40, among which, motif with 5 reiterations were the highest in frequency, followed by six, seven and eight repetition indicating that there is an inverse relationship between number of reiteration of a SSR motifs and its frequency. To survey the trend of distribution of SSR loci in candidate gene sequences, the location of motifs were thoroughly investigated. Our results showed that maximum percentage of SSR loci were found in CDS (37.22%) followed by 5′UTR (26.11%), intron (21.66%) and 3′UTR (15%) (Figure 1B). We classified the all 106 candidate genes into seven broad functional groups. Among the groups, 28.3% of total cgSSRs were found in transcription factor genes, while antioxidant genes contained 9.43% of total cgSSRs (Figure 1C). Further we have analyzed the location of SSR loci in each individual functional group. The result revealed that most SSR loci were found in CDS region in case of transcription factor genes and of genes involved in DNA/RNA modification and in intronic region in case of catalytic and antioxidant genes, whereas the genes showing kinase activity and involved in signaling showed highest frequency of SSRs in 5′UTR (Figure 1D). Equal percentage of SSR loci were found in CDS and intron of transporter genes (Figure 1D). Although salt responsive cgSSRs are present on all 12 chromosomes of rice, yet their distribution were not equal among the chromosomes (Figure 2). For example, maximum frequency (23.88%) of salt responsive cgSSR loci were found on chromosome 1, whereas the least (2.22%) was found on chromosome 10. Chromosome 2, 3 and 5 were found to contain more than 10% cgSSR loci.

### Development and validation of salt responsive candidate gene based SSR (cgSSR) markers

**Figure 3:**
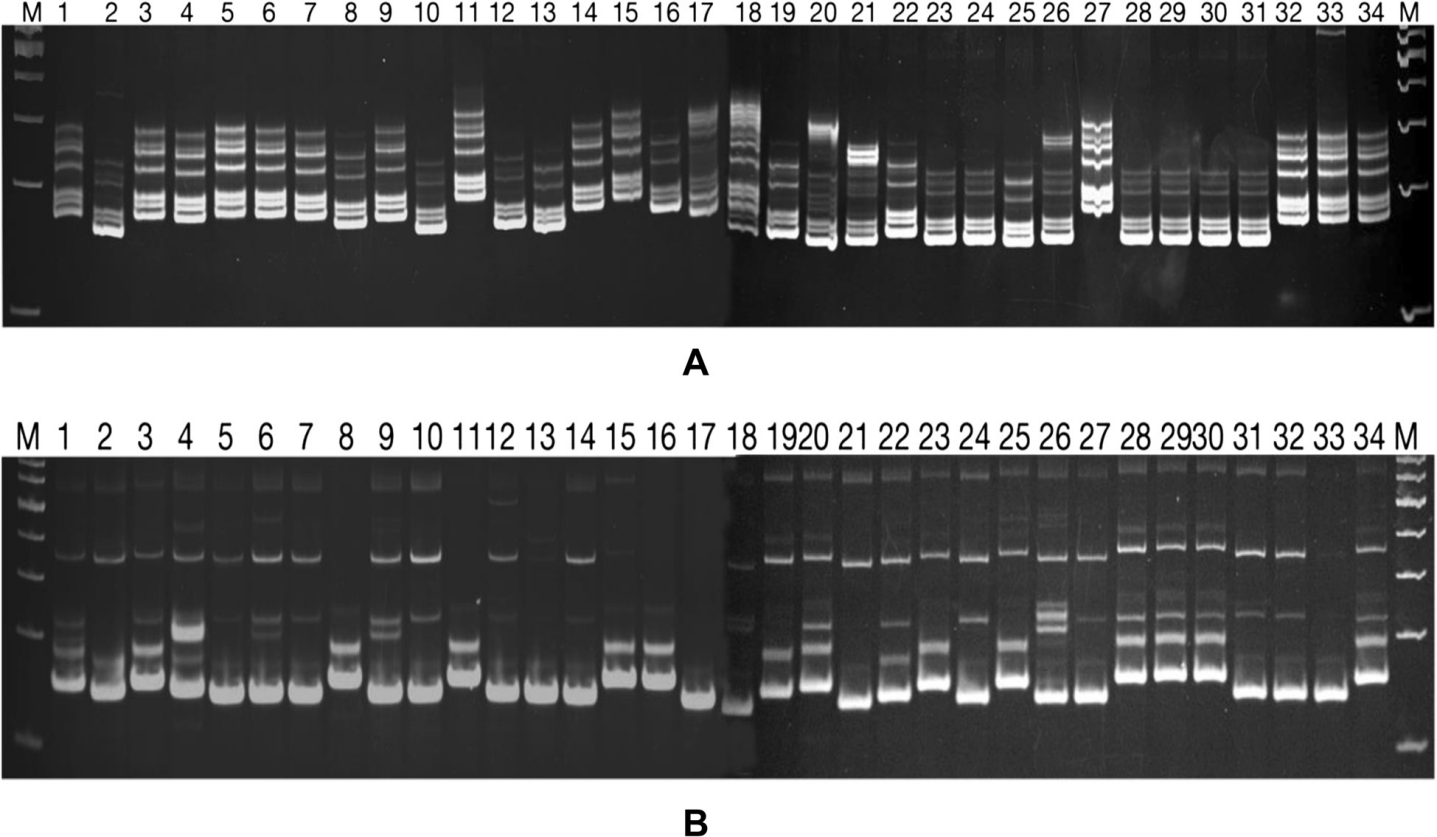
Representative images of 6% Polyacrylamide gel profile of amplified product from 34 genotypes using salt responsive cgSSR primer. **A**- Gel picture with marker- OsRacB (2)-SSR and **B**- with marker OsCML11-SSR. Image was taken in gel documentation system after staining with EtBr. Lane M- 100 bp DNA ladder (Thermo scientific), 1-34- different rice genotypes as defined in Additional file 1.

Out of 180 cgSSRs, primers were designed for 19 loci (NCBI probe- Pr032302526- Pr032302544) from 17 different salt responsive candidate genes (Table 1) for validation. Among 19 different loci, only one designed from CML31 gene failed to amplify. Therefore, we used finally 18 different cgSSR loci to study polymorphism in 34 rice genotypes containing two contrasting panels (17 tolerant and 17 susceptible genotypes). All 18 primer sets generated clear distinct polymorphic profiles as evident from the 6% agarose gel profile (Figure 3) and PIC values. A total of 393 alleles were detected including 32 rare alleles and 32 unique alleles. The average number of alleles produced per primer was 21.8. There was also a degree of stutter bands associated with the main alleles of almost half of the markers used. The cgSSR from *Nac5* gene produced the lowest number of alleles (10), whereas the cgSSR from *CML11* gave rise to the highest number (42) of alleles. Although SSR markers are multiallelic in nature, in order to avoid erroneous calculation and to ascertain the nature of amplicons, we have sequenced all the amplified bands for a particular genotype with a specific marker as a representative case. A total of 20 randomly chosen alleles were sequenced and aligned with the original target sequence. The alignment results confirmed the similarity of each bands with its particular original target sequence (data not shown).

The PIC value denotes the allelic diversity and frequency among genotypes. In our study, an average of about 0.278 PIC value was obtained per cgSSR. The lowest PIC value (0.087) was exhibited by the cgSSR from *UGE1* gene, while highest value (0.386) was obtained with the cgSSR from tri-nucleotide motif of *RacB* gene. Primer designed from di-nucleotide motif loci of *RacB* had a bit lower PIC value of 0.318. On the contrary, cgSSR primers based on di-nucleotide and tri-nucleotide motif loci of the gene *CAX* showed a PIC value of 0.326 and 0.169 respectively. Details of primers and their corresponding PIC values were depicted in the Table 1.

### Genetic diversity analysis using salt responsive cgSSR

**Figure 4:**
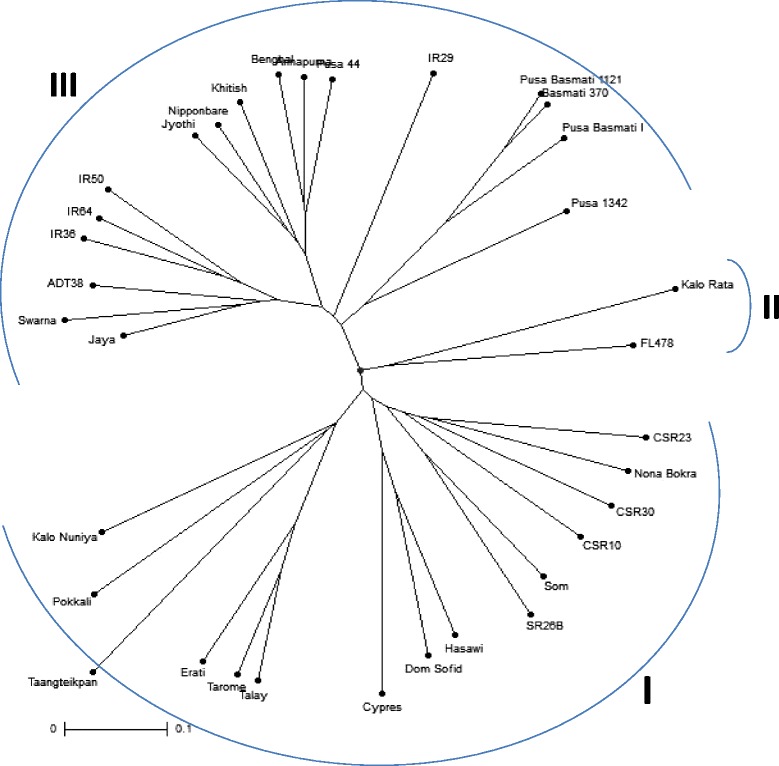
Dendrogram generated from an unweighted pair group method analysis (UPGMA) cluster analysis s based on salt responsive cgSSR markers. First two clusters showing all tolerant genotypes, whereas third cluster showing all susceptible genotypes.

The data matrix generated from 18 cgSSRs profiling of 34 genotypes were utilized to study the genetic diversity by dissimilarity analysis, factorial analysis through PCoA (principal coordinates analysis) and cluster analysis. The dendrogram generated through unweighted pair group method of arithmetic mean (UPGMA) showed the similarity among the rice accessions ranging from 19% to 89%. The dendrogram exhibited 3 distinct clusters of which two containing all salt tolerant genotypes and one single cluster containing all susceptible rice genotypes (Figure 4). The salt tolerant genotypes were more diverse than the salt susceptible panel in our study. Cluster I consisted of 15 tolerant genotypes containing 2 sub clusters (IA and IB). IA sub-cluster contained 6 genotypes, viz. two Indian- Kalo Nuniya, Pokkali and 4 exotic- Taangteikpan, Erati, Tarome and Talay, while IB sub-cluster contained 9 genotypes, viz. 4 exotic- Cypress, Dom Sofid, Hasawi, Som and 5 Indian- SR26B, CSR10, CSR30, CSR23 and Nona Bokra. Interestingly the smallest cluster (cluster II) contains exclusively two salt tolerant genotypes FL478 and Kala Rata. On the other hand, the largest cluster (cluster III) incorporated all salt susceptible genotypes in the study. Interestingly, all aromatic basmati rice genotypes (Pusa Basmati 1121, Pusa basmati I and Basmati 370) were grouped closely in the same sub cluster. Similarly, IR36, IR64 and IR50 were clustered together. Hence, it is distinct from the genetic diversity analysis using the 18 cgSSR markers that those markers are able to distinguish rice genotypes on the basis of salt sensitivity.

**Figure 5:**
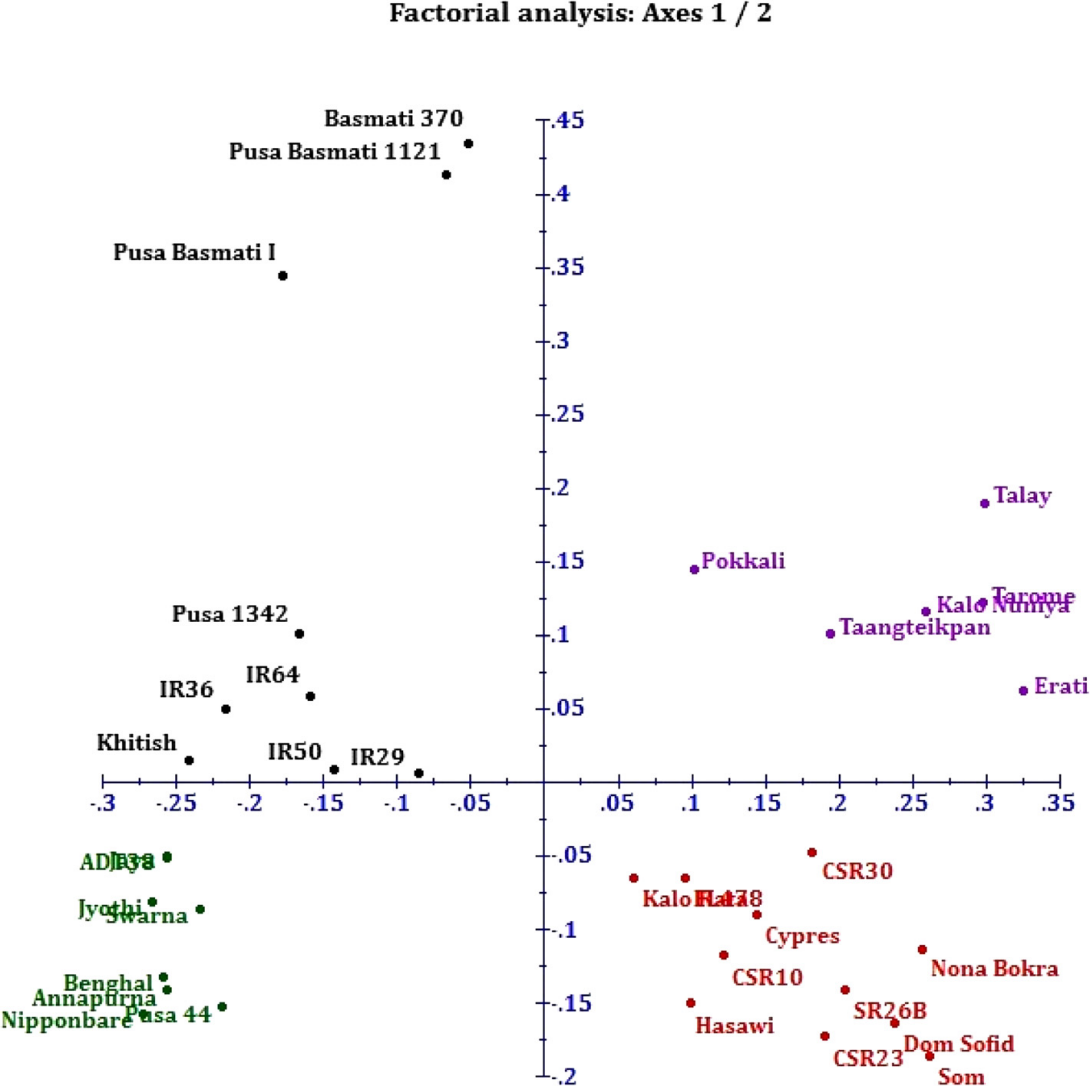
Two-dimension plot generated from principal coordinate analysis (PCoA) for all 34 rice genotypes. Red and violet colour was used for salt tolerant genotypes, while black and green colour was used for salt sensitive genotypes.

For overall representation of diversity, principal coordinates analysis (PCoA) which requires Euclidean distance between units has been performed. PCoA revealed distinct separation between each two rice genotypes (Figure 5). In accordance with the dendrogram, the PCoA also clearly divided the susceptible and tolerant panel without a single intermixing. Despite of being its one of the parent, susceptible IR29 was grouped separately from tolerant FL478. In a similar fashion, susceptible parent Jaya was in different group from the tolerant descendant CSR10. So it is clear that the developed cgSSRs from salt responsive genes distinguish the genotypes on account of their behavior in salt stress.

## Discussion

Ubiquitously, no toxic substance restricts plant growth more than does salt [35]. Salt stress is an emerging threat not only to rice but also to all glycophytes. Salinity has a tremendous effect on plant growth and reproduction as it imposes two simultaneous stresses- one in the form of toxic salt ions and other in the form of water stress caused by a certain drop in water potential value of the soil solution. Although a majority of rice abiotic stress biologists focused on deciphering the mechanism, developing resistance and identifying candidate genes and QTL involved in salt stress, yet, very few salt tolerant commercially available varieties have been developed. In order to enrich the genomic resource for developing salinity tolerance in rice, here we report the development of salt responsive candidate gene based SSR markers (cgSSRs) in rice for the first time. However, in maize, SSR markers were identified from genes involved in zinc and iron transporter [26] and from candidate genes related to tryptophan and lysine metabolic pathways [27]. Unlike the previous reports, all types of characterized candidate genes including transporter, transcription factor, antioxidant, DNA/RNA modifying which showed differential regulation under salinity stress in rice were selected from published literature and their sequence were used to mine SSR loci. Result of our study showed that tri-nucleotide repeats (56.11%) are more abundant than di- (42.11%) and tetra- (2.8%) nucleotide repeats which is in accordance with previously published reports on rice SSR [17,36] and common bean genic SSR [22]. Similar kind of result was demonstrated in an *in silico* analysis of cereals (rice, wheat, maize, barley, oat and rye) EST derived SSRs showing tri-nucleotide were the most frequent (54–78%) followed by di- (17.1–40.4%) and tetra- (3–6%) nucleotide [37]. However, contrastingly, it has been reported that the number of tri-nucleotide repeats was lesser than the number of di-nucleotide repeats in rice genic non coding microsatellites (GNMS) [38]. Most of the tri-nucleotide motifs were found in CDS (59%) followed by in 5′UTR (21%), intron (11%) and 3′UTR (9%). In this respect, the result of our study is in agreement with an earlier report in wheat [39]. Other studies in rice also support our finding of highest frequency of occurrence of tri-nucleotide repeats in CDS region than any other region like 5′UTR, intron and 3′UTR [38,40]. The phenomenon of copiousness of tri-nucleotide repeats in CDS could be attributed to the selection pressure against frame shift mutation in coding regions resulting from length changes in nontriplet repeats [41]. A previous study of Fujimori et al. [40] proposed that there is a gradual reduction of microsatellite density along the direction of transcription in plant. However, in our study, except the highest frequency in CDS, microsatellite density declines along the direction of transcription (5′UTR—›Intron—›3′UTR) (Figure 1B). In our study, arginine coding (CGG) and alanine coding (GCC) tri-nucleotide repeat motifs were found as two most abundant classes which is in accordance of a previous study of unigene derived microsatellites in cereals [42].

Keeping in mind to validate those salt responsive cgSSRs, we analyzed their possible role to distinguish salt tolerant and susceptible rice genotypes. We speculated the repeat length variations in those cgSSR loci may play role in the manifestation of differential behavior of rice genotypes to salt stress. In order to demonstrate experimental evidence on the speculation, 19 selected cgSSRs were tested in two contrasting panels of rice genotypes which differ in salt sensitivity. The selection of those cgSSRs was based on the notion that SSR loci with more repeats tend to be more polymorphic [43]. SSR loci with 9 or more repeats have been selected to study polymorphism. With the exception of one which failed to amplify, remaining all 18 cgSSRs exhibited polymorphic banding pattern supporting our speculated hypothesis regarding the high level of diversity in salt responsive genes. Among the 18 cgSSRs, six was comprised of tri-nucleotide motif and twelve was with di-nucleotide motif (Table 1). As evidenced from PIC value, polymorphism level varies from primer to primer. Usually di-nucleotide repeats containing SSRs are more prone to mutation and as a result they show more polymorphism than tri-nucleotide repeats containing SSRs [43,44]. However, in our study, the mean difference of PIC value between di-nucleotide and tri-nucleotide containing cgSSRs was not quite statistically significant (*p* value 0.0627). The mean PIC value of all 18 cgSSR primers in the present study was 0.278 which is higher than the previous report describing salt responsive miRNA-SSR markers in rice [31]. Nevertheless, higher PIC values for SSR primers from genomic sequences of rice were reported in earlier studies [7,45]. This might be due to the fact that genic SSRs usually reveal less polymorphism in comparison with genomic SSRs as reviewed in a previous report [16]. The average number of alleles per locus was 21.8 in the present study. This average value is higher in comparison with the average value published in earlier reports [7,27,46,47]. However, producing more alleles than the presence of its repeats by SSR markers is also well documented in literature [48,49] which corroborate our present findings.

It is noteworthy of the present study that the 18 cgSSR markers were remarkably capable of indicating the variation or diversity among rice genotypes in relation to their salinity responsive characters. In the present study, dendrogram generated by UPGMA clearly established relationship between different rice genotype according to their salt sensitivity (Figure 4). Of the three clusters generated in the dendrogram, two contained all tolerant genotypes and another one was comprised of all salt susceptible genotypes exclusively. Our result is also in accordance with the result about the similar clustering pattern of Nona Bokra and Pokkali [50], CSR23, CSR10 and SR 26B [31], Hasawi and SR26B [51] and susceptible IR36 and IR64 [52]. Cluster II was consisted of two tolerant genotypes FL478 and Kala Rata. Similar grouping was observed in the report of salt responsive miRNA-SSR [31]. Remarkably, no single genotype from a particular panel (salinity tolerant or susceptible) is intermixed with another panel. However, out-grouping and intermixing of quite a few salt tolerant and susceptible genotypes were reported previously [31,53,54]. The similarity value between genotypes in the present study ranged from 19% to 89%. In this regard, our result is comparable to the reports published previously [55]. Of all 18, only one cgSSR, CML11 is located at a nearby position (17.58 Mb) of the well known major QTL Saltol (10.8-16.4 Mb) on chromosome 1 indicating it’s possibility of being used in MAS programme [56]. Microsatellite within genes can play vital role in gene regulation for controlling a particular trait. SSR in CDS can lead to a gain or loss of gene function via frameshift mutation, SSR in 5′-UTRs can affect transcription and translation, SSR in 3′-UTRs can disrupt splicing and, possibly, disrupt other cellular function and intronic SSRs can affect gene transcription and mRNA splicing [23]. The diversity analysis in the study was based on cgSSR markers representing all classes (CDS, 5′UTR, 3′UTR and intronic) (see Table 1). As no null alleles were obtained, the possibility of the presence or absence of a particular allele in contrasting genotypes is discarded. Hence, our result clearly provoked a thought that the variation present in the salt responsive gene’s microsatellite loci may be a key role player in the behavioral response of rice genotypes to salinity. The variation may also play important role in the differential molecular regulation of those genes in rice which differs in salt sensitivity.

## Conclusion

To conclude, the present study represents an extensive identification of salt responsive candidate gene based SSR (cgSSR) and their validation as a remarkable tools to distinguish salt susceptible and salt tolerant rice genotypes. The cgSSRs developed here distinctly demarcated the distance between rice genetic resources which show different response to salinity. Identification of these types of allelic variations within salt responsive candidate genes from contrasting panel can provide unique genomic resources with delivering novel alleles to develop improved varieties for salt tolerance. Those developed cgSSRs markers have high potential of linkage and can be utilized for gene pyramiding in breeding programme for salt tolerance trait in rice. They may be proved as an aid in robust functional diversity analysis in the available array of rice germplasms and also in natural population. As there is a high chance of being conserved in nature, the cgSSRs markers are hypothesized to be highly transferable to other cereals which also face tremendous yield losses from salinity. To insight the exact molecular mechanism of that variation in the microsatellite loci governing different sensitivity to salinity, further intense investigation is required.

### Availability of supporting data

Salt tolerance ad susceptible panel of rice germplasms used for validation of cgSSR markers are available in Additional file 1. Name of all 106 salt responsive genes, LOC number, number and position of SSR and their respective references are available in Additional file 2. All 19 cgSSR marker used in the present study to construct phylogenetic tree can be found as NCBI probe- Pr032302526- Pr032302544. The phylogenetic tree for the study have been submitted to DRYAD http://datadryad.org/review?doi=doi:10.5061/dryad.dj51c.

## Additional files

Additional file 1:
**Rice genotypes used in diversity analysis using salt responsive cgSSR markers.**
Additional file 2:
**Salt responsive genes with their LOC number, function and their number, locations.**
Additional file 3:
**Bar diagram showing frequency of different types of cgSSR motifs found in salt responsive candidate genes of rice.**
