# Supplementary material for: Identification and analysis of novel salt responsive candidate gene based SSRs (cgSSRs) from rice (Oryza sativa L.)
Source: BMC Plant Biol. 2015 May 16;15:122. doi: 10.1186/s12870-015-0498-1 (PMC4435636; doi:10.1186/s12870-015-0498-1)
Supplement: Additional file 1: — Rice genotypes used in diversity analysis using salt responsive cgSSR markers. [file 12870_2015_498_MOESM1_ESM.docx]

**Additional file 1**: Details of rice germplasms used in diversity analysis using salt responsive cgSSR matrkers

| **Rice germplasm** | **Salt sensitivity** | **Country of origin** | **Reference** |
| --- | --- | --- | --- |
| Pokkali | Tolerant | India | [1] |
| FL478 | Tolerant | Philippines | [2] |
| Kalo Nuniya | Tolerant | India | [3] |
| Kala Rata | Tolerant | India | [1] |
| Nona Bokra | Tolerant | India | [1] |
| CSR10 | Tolerant | India | [4] |
| CSR23 | Tolerant | India | [5] |
| CSR30 | Tolerant | India | [6] |
| SR26B | Tolerant | India | [2] |
| Som | Tolerant | Guinea-Bissau | [1] |
| Eratio | Tolerant | Portugal | [1] |
| Tarome | Tolerant | Iran | [7] |
| Talay | Tolerant | Myanmar | [1] |
| Cypres | Tolerant | USA | [8] |
| Dom Sofid | Tolerant | Iran | [1] |
| Hasawi | Tolerant | Saudi Arabia | [1] |
| Taangteikpan | Tolerant | Myanmar | [1] |
| IR29 | Susceptible | Philippines | [1] |
| IR50 | Susceptible | Philippines | [9] |
| IR64 | Susceptible | Philippines | [10] |
| IR36 | Susceptible | Philippines | [3] |
| Swarna | Susceptible | India | [11] |
| Jaya | Susceptible | India | [4] |
| Nipponbare | Susceptible | Japan | [1] |
| Jyothi | Susceptible | India | [4] |
| Khitish | Susceptible | India | [12] |
| ADT38 | Susceptible | India | [9] |
| Annapurna | Susceptible | India | [13] |
| Benghal | Susceptible | India | [14] |
| Pusa 1342 | Susceptible | India | [4] |
| Pusa 44 | Susceptible | India | [4] |
| Pusa Basmati I | Susceptible | India | [11] |
| Basmati 370 | Susceptible | India | [15] |
| Pusa Basmati 1121 | Susceptible | India | [4] |

References

1. Platten JD, Egdane JA, Ismail AM: **Salinity tolerance, Na+ exclusion and allele mining of HKT1; 5 in Oryza sativa and O. glaberrima: many sources, many genes, one mechanism?** *BMC plant biology* 2013, **13**(1):32.

2. Islam MR, Gregorio GB, Salam MA, Collard BC, Singh RK, Hassan L: **Validation of SalTol linked markers and haplotype diversity on chromosome 1 of rice**. *Molecular Plant Breeding* 2012, **3**(1).

3. Ganie SA, Karmakar J, Roychowdhury R, Mondal TK, Dey N: **Assessment of genetic diversity in salt-tolerant rice and its wild relatives for ten SSR loci and one allele mining primer of salT gene located on 1st chromosome**. *Plant Systematics and Evolution* 2014, **300**(7):1741-1747.

4. Singh A, Singh PK, Singh R, Pandit A, Mahato AK, Gupta DK, Tyagi K, Singh AK, Singh NK, Sharma TR: **SNP haplotypes of the BADH1 gene and their association with aroma in rice (Oryza sativa L.)**. *Molecular breeding* 2010, **26**(2):325-338.

5. Mishra B, Singh R, Senadhira D, Khush G, Brar D, Hardy B: **Advances in breeding salt tolerant rice varieties**. In: *Advances in Rice Genetics Supplement to Rice Genetics IV Proceedings of the Fourth International Rice Genetics Symposium, 22–27 October 2000: 2003*. Los Banos Philippines: 5-7.

6. Seetharam K, Thirumeni S, Paramasivam K: **Estimation of genetic diversity in rice (Oryza sativa L.) genotypes using SSR markers and morphological characters**. *African Journal of Biotechnology* 2009, **8**(10).

7. Ahmadi J, Fotokian M-H: **Identification and mapping of quantitative trait loci associated with salinity tolerance in rice (Oryza Sativa) using SSR markers**. *Iranian Journal of Biotechnology* 2011, **9**(1).

8. Iyer S, Caplan A: **Products of Proline Catabolism Can Induce Osmotically Regulated Genes in Rice**. *Plant Physiology* 1998, **116**(1):203-211.

9. Djanaguiraman M, Senthil A, Ramadass R: **Mechanism of salt tolerance in rice genotypes during germination and seedling growth**. *Indian Journal of Agricultural Research* 2004, **38**(1):73-76.

10. Sarhadi E, Bazargani MM, Sajise AG, Abdolahi S, Vispo NA, Arceta M, Nejad GM, Singh RK, Salekdeh GH: **Proteomic analysis of rice anthers under salt stress**. *Plant Physiology and Biochemistry* 2012, **58**:280-287.

11. Basu S, Roychoudhury A, Saha P, Sengupta D: **Comparative analysis of some biochemical responses of three indica rice varieties during polyethylene glycol-mediated water stress exhibits distinct varietal differences**. *Acta Physiol Plant* 2010, **32**(3):551-563.

12. Ganguly M, Datta K, Roychoudhury A, Gayen D, Sengupta DN, Datta SK: **Overexpression of Rab16A gene in indica rice variety for generating enhanced salt tolerance**. *Plant signaling & behavior* 2012, **7**(4):502-509.

13. Swapna T: **Salt stress induced changes on enzyme activities during different developmental stages of rice (Oryza sativa Linn.)**. *Indian Journal of Biotechnology* 2003, **2**:251-258.

14. Karan R, DeLeon T, Biradar H, Subudhi PK: **Salt Stress Induced Variation in DNA Methylation Pattern and Its Influence on Gene Expression in Contrasting Rice Genotypes**. *PLoS ONE* 2012, **7**(6):e40203.

15. Basu S, Gangopadhyay G, Mukherjee B, Gupta S: **Plant regeneration of salt adapted callus of indica rice (var. Basmati 370) in saline conditions**. *Plant Cell, Tissue and Organ Culture* 1997, **50**(3):153-159.
