# Supplementary material for: Identification and analysis of novel salt responsive candidate gene based SSRs (cgSSRs) from rice (Oryza sativa L.)
Source: BMC Plant Biol. 2015 May 16;15:122. doi: 10.1186/s12870-015-0498-1 (PMC4435636; doi:10.1186/s12870-015-0498-1)
Supplement: Additional file 2: — Salt responsive genes with their LOC number, function and their number, locations. [file 12870_2015_498_MOESM2_ESM.doc]

| **Sl. no** | **Gene name** | **LOC No.** | **Function** | **(Motif)repeats** | **Location in gene sequence** | **Reference** |
| --- | --- | --- | --- | --- | --- | --- |
| 1 | OsSIK1 | LOC_Os06g03970 | Signalling, Kinase | (CT)6 and (AG)6 | 5'UTR and 5'UTR | [1] |
| 2 | OsSNAC1 | LOC_Os03g60080 | TF | (CG)5 and (GCC)6 | 3'UTR and CDS | [2] |
| 3 | OsCOIN | LOC_Os01g01420.1 | RING finger TF | (CGA)6 and (GATA)5 | CDS and 5'UTR | [3] |
| 4 | OsNAC5 | LOC_Os11g08210 | TF | (TAA)18 | 3’UTR | [4] |
| 5 | OsBIHD1 | LOC_Os03g47740 | TF (HD) | (GA)5 and (CT)7 | 5'UTR and 5'UTR | [5] |
| 6 | OsSKC1 | LOC_Os01g20160.1 | Functional, ion-transporter | (TA)5 and (GCA)6 | Intronic and CDS | [6] |
| 7 | OsSOS2 | LOC_Os06g40370 | Signalling, Kinase | (CT)7 | Intronic | [7] |
| 8 | OsDREB1A | LOC_Os09g35030 | DNA binding protein | (AC)5, (GC)5 and (CGG)6 | 5'UTR, CDS and CDS | [8] |
| 9 | OsTPS1 | LOC_Os05g44210 | Functional, Trehalose biosynthesis | (GC)5 and (TCC)5 | 5'UTR and CDS | [9] |
| 10 | OsRab7 | LOC_Os05g44050 | Signalling GTP binding | (CCT)5 | 5'UTR | [10] |
| 11 | ONAC045 | LOC_Os11g03370 | TF | (CGA)5 and (TGC)6 | Intronic and 3'UTR | [11] |
| 12 | OsZFP182 | LOC_Os03g60560 | TF | (CGG)5 | CDS | [12] |
| 13 | OsZFP252 | LOC_Os12g41660 | TF | (GGC)6, (CCT)6, (CCG)7, (GCC)5 and (ACT)5 | CDS, CDS, CDS, CDS and 3'UTR | [13] |
| 14 | OsSNAC2 | LOC_Os01g66120 | TF | (GT)5 | 3'UTR | [14] |
| 15 | OsDST | LOC_Os03g57240 | TF, H2O2-homeostasis | (GCC)5, (CGG)5 and (CCG)7 | CDS, CDS and 3'UTR | [15] |
| 16 | OsTZF1 | LOC_Os05g10670 | TF,ZF | (GT)5, (GCG)5, (CCG)6 and (CGC)6 | 3'UTR, CDS, CDS and CDS | [16] |
| 17 | OsWRKY45 | LOC_Os05g25770 | TF | (GCG)7 | CDS | [17] |
| 18 | OsNAC6 | LOC_Os01g66120 | TF | (GT)5 | 3'UTR | [18] |
| 19 | OsCIPK15 | LOC_Os11g02240 | Signalling | (CT)6 | 5'UTR | [19] |
| 20 | OsCam1-1 | LOC_Os03g20370 | Signalling | (GAA)5 | 5'UTR | [20] |
| 21 | OsCML5 | LOC_Os12g41110 | Signalling | (TCT)7 | 5'UTR | [21] |
| 22 | OsMyb3R-2 | LOC_Os01g62410 | TF | (GTG)7 and (GGC)5 | 5'UTR and CDS | [22] |
| 23 | OsMyb2 | LOC_Os03g20090 | TF | (GC)7, (GCA)5 and (CATC)5 | 3'UTR, intronic and 5'UTR | [23] |
| 24 | OsCPK-21 | LOC_Os08g42740.3 | Signalling, Kinase | (TC)11 and (GGA)5 | Intronic and CDS | [24] |
| 25 | OsBIERF3 | LOC_Os02g43790 | TF | (GGA)5 and (GCC)5 | CDS and CDS | [25] |
| 26 | OsERF922 | LOC_Os01g54890 | TF | (GTC)7 | CDS | [26] |
| 27 | OsHsfA7 | LOC_Os01g39020 | TF, Chaperon | (TCC)7 | CDS | [27] |
| 28 | OsDREB1F | LOC_Os01g73770.1 | TF | (GC)5 and (CG)5 | CDS and CDS | [28] |
| 29 | OsAKT1 | LOC_Os01g45990 | Functional, Ion transport | (CGG)5 | CDS | [29] |
| 30 | OrbHLH001 | LOC_Os01g70310.1 | TF | (GGC)6, (GGA)5 and (CCA)8 | CDS, CDS and CDS | [30] |
| 31 | OrbHLH2 | LOC_Os11g32100 | TF | (GGT)5 and (CGG)6 | CDS and CDS | [31] |
| 32 | OsECS | LOC_Os05g03820.3 | Functional, antioxidation | (TA)6 and (GGA)5 | 3'UTR and CDS | [32] |
| 33 | H+- ATPase/OSA3 | LOC_Os12g44150.1 | Functional,Ion-transporter | (TC)7 and (CA)6 | Intronic and intronic | [33] |
| 34 | OsCBSX4 | LOC_Os03g52690 | Regulatory, Adenosine binding | (TCC)7 | 5'UTR | [34] |
| 35 | OsP5CR | LOC_Os01g71990 | Functional, Osmoprotection | (CCT)8 | 5'UTR | [35] |
| 36 | OsiSAP8 | LOC_Os06g41010.1 | Signalling, protein binding | (AT)7 | 5'UTR | [36] |
| 37 | OsHsfC1b | LOC_Os01g53220 | TF, chaperon | (GA)5 | 3'UTR | [37] |
| 38 | OsSERF1 | LOC_Os05g34730 | TF, Inhibition of MAPK cascade | (CCG)5 | CDS | [38] |
| 39 | Osr40c1 | LOC_Os03g21040.2 | ABA responsive protein | (CGG)6 | CDS | [39] |
| 40 | OsGMST1 | LOC_Os02g17500 | Functional, Sugar transporter | (AG)10 | 5'UTR | [40] |
| 41 | OsCPK17 | LOC_Os07g06740.2 | Signalling, Kinase | (CT)9 and (CT)11 | 3'UTR and 3'UTR | [41] |
| 42 | OsAOX1a | LOC_Os04g51150.1 | Functional, electron transport | (CGG)8 | CDS | [42] |
| 43 | OsAOX1b | LOC_Os04g51160 | Functional, electron transport | (CGG)5 | CDS | [42] |
| 44 | OsCAX | LOC_Os02g04630 | Functional, Ion-channel | (CT)18 and (TTA)26 | Intronic and Intronic | [43] |
| 45 | OsTPC1 | LOC_Os01g48680 | Functional, Ion-channel | (GCG)5 and (TTA)5 | CDS and intron | [43] |
| 46 | OsBADH1 | LOC_Os04g39020.1 | Functional, Osmoprotection | (AGC)5 | 5'UTR | [44] |
| 47 | OsC3H33 | LOC_Os05g03760 | Regulatory, RNA processing | (CG)5, (CGC)5 and (GGC)6 | CDS, CDS and CDS | [45] |
| 48 | OsC3H37 | LOC_Os05g45020 | Regulatory, RNA processing | (ATT)5 | 3'UTR | [45] |
| 49 | OsC3H50 | LOC_Os07g38090 | Regulatory, RNA processing | (TC)9 | 5'UTR | [45] |
| 50 | OsHKT8 | LOC_Os01g20160 | Functional, Ion transport | (TA)5 and (GCA)6 | Intronic and CDS | [46] |
| 51 | OSMT1e-P | LOC_Os11g47809.1 | Cysteine-rich, metal binding | (GT)8 | 3'UTR | [47] |
| 52 | OsTIFY11a | LOC_Os03g08310 | Regulatory | (GCC)7 | CDS | [48] |
| 53 | OsWRKY-13 | LOC_Os01g54600 | TF | (GA)9 and (AG)16 | 5'UTR and 5'UTR | [49] |
| 54 | OsCML8 | LOC_Os10g25010 | Regulatory, Ca2+-binding | (CGG)9 | CDS | [50] |
| 55 | OsCML11 | LOC_Os01g32120 | Regulatory, Ca2+-binding | (CAG)10 | CDS | [50] |
| 56 | OsCML31 | LOC_Os01g72530.1 | Regulatory, Ca2+-binding | (AT)40 | 3'UTR | [51] |
| 57 | OsABP | LOC_Os06g33520 | Regulatory, Helicase | (TA)5 | CDS | [52] |
| 58 | OsSKIPa | LOC_Os02g52250 | Regulatory, Spliceosome component | (GA)5 and (GCG)6 | CDS and CDS | [53] |
| 59 | OsMIOX | LOC_Os06g36560.1 | Balances the concentration of myo-inositol | (TC)6 and (CGG)5 | Intronic and CDS | [54] |
| 60 | OsCA1 | LOC_Os01g45274.1 | Chloroplast carbonic anahydrase | (CT)5,(CG)5,(CT)9,(CCG)6,(TCC)7,(AGC)6 and(CTG)5 | Intronic, intronic, intronic, CDS, CDS, intronic and intronic | [55] |
| 61 | OsAPXb | LOC_Os07g49400.2 | Functional, Antioxidation | (TTC)6 | Intronic | [56] |
| 62 | OsPUB15 | LOC_Os08g01900 | Functional, ubiquitination | (TCC)5 | 5'UTR | [57] |
| 63 | OsRacB | LOC_Os02g02840.1 | Signalling, GTPase | (GA)21,(TTC)9,(TTC)5 | 5'UTR, intronic and intronic | [58] |
| 64 | OsSDIR1 | LOC_Os03g16570.2 | Functional, ubiquitination | (GTG)7 | 5'UTR | [59] |
| 65 | OsUGE-1 | LOC_Os05g51670.1 | Functional, nucleotide sugar interconversion | (TC)9,(AT)8,(AT)6,(AGC)5,(TAC)6,(CTTC)5,(TCGG)5 | Intronic, intronic, intronic, 3'UTR, 3'UTR, intronic, intronic | [60] |
| 66 | OsWNK1 | LOC_Os07g38530.1 | Signalling, Kinase | (AGC)6 | 5'UTR | [61] |
| 67 | OsHsp90 | LOC_Os06g50300.1 | Functional, Heat shock | (CTC)8 | CDS | [62] |
| 68 | OsGR3 | LOC_Os10g28000 | Functional, Reduces GSSG | (AT)5 | Intronic | [63] |
| 69 | OsMGD | LOC_Os02g55910.1 | Functional, Lipid Biosynthesis | (TC)7,(CGT)7 | 5'UTR and CDS | [64] |
| 70 | OsMSRMK3 | LOC_Os06g48590.1 | Signalling, Kinase | (CT)12,(CT)5,(TCC)7 | 5'UTR, 5'UTR and 5'UTR | [65] |
| 71 | OsTOP6A3 | LOC_Os03g17610.1 | Functional, Topoisomerase | (GTC)7 | CDS | [66] |
| 72 | OsCDKC;1 | LOC_Os01g72790.1 | Regulatory, Kinase | (TC)5 | Intronic | [67] |
| 73 | OsCLC-1 | LOC_Os02g35190.2 | Functional, Ion transport | (CA)5,(AGA)11,(GCG)5 | 5'UTR, 5'UTR and 5'UTR | [68] |
| 74 | OsGGT | LOC_Os10g40640.1 | Functional, Glycosylation | (TC)5,(CTA)6 | Intronic and 5'UTR | [69] |
| 75 | OsOXHS2 | LOC_Os01g03570 | Regulatory, protein-protein interaction | (CCG)5,(CCA)6 | 5'UTR and CDS | [70] |
| 77 | OsbZIP71 | LOC_Os09g13570 | TF | (TA)12 and (AT)7 | 5'UTR and intronic | [71] |
| 78 | OsMAPK44 | LOC_Os08g06060.1 | Signalling, Kinase | (GGC)6 and (CGG)5 | CDS and CDS | [72] |
| 79 | OsDSM1 | LOC_Os02g50970 | Signalling, Kinase | (CGG)7 | 5'UTR | [73] |
| 80 | OsAPX4 | LOC_Os08g43560.1 | Functional, anti-oxidation | (TA)5 | Intronic | [74] |
| 81 | OsglyII | LOC_Os09g34100.1 | Functional, glyoxalate-pathway | (GCG)5 | 5'UTR | [75] |
| 82 | OsMSRB | LOC_Os06g27760 | Functional, antioxidation | (TCG)6 | CDS | [76] |
| 83 | Osmyb4 | LOC_Os04g43680.1 | TF | (GCC)5 | CDS | [77] |
| 84 | OsNOA1 | LOC_Os02g01440 | Functional, NO synthesis | (AATC)5 | Intronic | [78] |
| 85 | OsPEX11-1 | LOC_Os03g02590 | Functional, peroxisomal biogenesis | (TG)19 | Intronic | [79] |
| 86 | OsSRWD2 | LOC_Os02g48964 | Regulatory, Chromatin modification,Transcription | (CG)5 | 5'UTR | [80] |
| 87 | OsSRWD3 | LOC_Os06g07540 | Regulatory, Chromatin modification,Transcription | (TA)8,(TC)5 | Intronic and intronic | [80] |
| 88 | OsSRWD4 | LOC_Os08g31560 | Regulatory, Chromatin modification,Transcription | (CGC)5 | Intronic | [80] |
| 89 | OsSRWD5 | LOC_Os03g26870 | Regulatory, Chromatin modification,Transcription | (AT)7, (AT)5 and (CGC)5 | Intronic, intronic and 3'UTR | [80] |
| 90 | OsSRZ1 | LOC_Os02g10920.4 | Regulatory, Splicing | (GCC)7 | 5'UTR | [81] |
| 91 | OsDBH1 | LOC_Os04g40970 | DEAD- box, ATP dependent RNA helicase | (TA)5 and (CCT)7 | 3'UTR and CDS | [82] |
| 92 | OsSAMDC | LOC_Os04g42090.4 | Functional, polyamine biosynthesis | (AT)5 | 3'UTR | [83] |
| 93 | OsCyP20–2 | LOC_Os05g01270 | Functional, Protein folding | (GA)6 | 5'UTR | [84] |
| 94 | OsAP21 | LOC_Os01g10370 | Ordered | (CGG)6 | CDS | [85] |
| 95 | OsAPX7 | LOC_Os04g35520 | Functional, antioxidation | (TG)6 | Intronic | [86] |
| 96 | OrbHLH2 | LOC_Os11g32100 | TF | (GGT)5 and (CGG)6 | CDS and CDS | [31] |
| 97 | OsACA6 | LOC_Os04g51610 | Ca2+ Atpase | (CT)10, (CA)8 and (GCG)5 | 3'UTR, intronic and CDS | [87] |
| 98 | OsHAP2E | LOC_Os03g29760 | TF | (AG)13, (GA)8 and (CAG)8 | 5'UTR, 5'UTR and CDS | [88] |
| 99 | OsHKT1;4 | LOC_Os04g51830 | Na+ transporter | (GGC)5 | CDS | [89] |
| 100 | OsHBP1b | LOC_Os01g17260 | TF | (CT)7, (TC)7 and (CCT)6 | 5'UTR, 5'UTR and 5'UTR | [90] |
| 101 | OsRINO1 | LOC_Os03g09250 | Myoinositol synthesis | (CCG)9 | 5'UTR | [91] |
| 102 | OsMYB48-1 | LOC_Os01g74410 | TF | (AT)6, (TG)5 and (ACG)5 | 3'UTR, 3'UTR and CDS | [92] |
| 103 | OsABCG5 | LOC_Os03g17350 | Transporter | (GAC)5 | 3'UTR | [93] |
| 104 | OsJAZ9 | LOC_Os03g08310 | Transcription regulation | (GCC)7 | CDS | [94] |
| 105 | OsrgMT | LOC_Os11g47809 | Metallothionein protein | (GT)8 | 3'UTR | [95] |
| 106 | OsPOP5 | LOC_Os02g18850 | Serine peptidases | (AG)6 | 3'UTR | [96] |

**Additional file 2: Details of salt responsive genes with their LOC number, function and motifs found. TF- transcription factor**

**Reference**

1. Ouyang SQ, Liu YF, Liu P, Lei G, He SJ, Ma B, Zhang WK, Zhang JS, Chen SY: **Receptor-like kinase OsSIK1 improves drought and salt stress tolerance in rice (Oryza sativa) plants**. *The Plant journal : for cell and molecular biology* 2010, **62**(2):316-329.

2. Saad ASI, Li X, Li H-P, Huang T, Gao C-S, Guo M-W, Cheng W, Zhao G-Y, Liao Y-C: **A rice stress-responsive NAC gene enhances tolerance of transgenic wheat to drought and salt stresses**. *Plant Science* 2013, **203**:33-40.

3. Liu K, Wang L, Xu Y, Chen N, Ma Q, Li F, Chong K: **Overexpression of OsCOIN, a putative cold inducible zinc finger protein, increased tolerance to chilling, salt and drought, and enhanced proline level in rice**. *Planta* 2007, **226**(4):1007-1016.

4. Takasaki H, Maruyama K, Kidokoro S, Ito Y, Fujita Y, Shinozaki K, Yamaguchi-Shinozaki K, Nakashima K: **The abiotic stress-responsive NAC-type transcription factor OsNAC5 regulates stress-inducible genes and stress tolerance in rice**. *Molecular Genetics and Genomics* 2010, **284**(3):173-183.

5. Luo H, Song F, Goodman R, Zheng Z: **Up‐Regulation of OsBIHD1, a Rice Gene Encoding BELL Homeodomain Transcriptional Factor, in Disease Resistance Responses**. *Plant Biology* 2005, **7**(5):459-468.

6. Ren ZH, Gao JP, Li LG, Cai XL, Huang W, Chao DY, Zhu MZ, Wang ZY, Luan S, Lin HX: **A rice quantitative trait locus for salt tolerance encodes a sodium transporter**. *Nat Genet* 2005, **37**(10):1141-1146.

7. Kumar G, Kushwaha HR, Purty RS, Kumari S, Singla-Pareek SL, Pareek A: **Cloning, structural and expression analysis of OsSOS2 in contrasting cultivars of rice under salinity stress**. *Genes Genomes Genomics* 2012, **6**:34-41.

8. Dubouzet JG, Sakuma Y, Ito Y, Kasuga M, Dubouzet EG, Miura S, Seki M, Shinozaki K, Yamaguchi-Shinozaki K: **OsDREB genes in rice, Oryza sativa L., encode transcription activators that function in drought-, high-salt- and cold-responsive gene expression**. *The Plant journal : for cell and molecular biology* 2003, **33**(4):751-763.

9. Li HW, Zang BS, Deng XW, Wang XP: **Overexpression of the trehalose-6-phosphate synthase gene OsTPS1 enhances abiotic stress tolerance in rice**. *Planta* 2011, **234**(5):1007-1018.

10. Nahm MY, Kim SW, Yun D, Lee SY, Cho MJ, Bahk JD: **Molecular and biochemical analyses of OsRab7, a rice Rab7 homolog**. *Plant & cell physiology* 2003, **44**(12):1341-1349.

11. Zheng X, Chen B, Lu G, Han B: **Overexpression of a NAC transcription factor enhances rice drought and salt tolerance**. *Biochemical and biophysical research communications* 2009, **379**(4):985-989.

12. Huang J, Sun S, Xu D, Lan H, Sun H, Wang Z, Bao Y, Wang J, Tang H, Zhang H: **A TFIIIA-type zinc finger protein confers multiple abiotic stress tolerances in transgenic rice (Oryza sativa L.)**. *Plant molecular biology* 2012, **80**(3):337-350.

13. Xu DQ, Huang J, Guo SQ, Yang X, Bao YM, Tang HJ, Zhang HS: **Overexpression of a TFIIIA-type zinc finger protein gene ZFP252 enhances drought and salt tolerance in rice (Oryza sativa L.)**. *FEBS Lett* 2008, **582**(7):1037-1043.

14. Hu H, You J, Fang Y, Zhu X, Qi Z, Xiong L: **Characterization of transcription factor gene SNAC2 conferring cold and salt tolerance in rice**. *Plant molecular biology* 2008, **67**(1-2):169-181.

15. Huang XY, Chao DY, Gao JP, Zhu MZ, Shi M, Lin HX: **A previously unknown zinc finger protein, DST, regulates drought and salt tolerance in rice via stomatal aperture control**. *Genes & development* 2009, **23**(15):1805-1817.

16. Jan A, Maruyama K, Todaka D, Kidokoro S, Abo M, Yoshimura E, Shinozaki K, Nakashima K, Yamaguchi-Shinozaki K: **OsTZF1, a CCCH-tandem zinc finger protein, confers delayed senescence and stress tolerance in rice by regulating stress-related genes**. *Plant Physiol* 2013, **161**(3):1202-1216.

17. Qiu Y, Yu D: **Over-expression of the stress-induced OsWRKY45 enhances disease resistance and drought tolerance in Arabidopsis**. *Environmental and Experimental Botany* 2009, **65**(1):35-47.

18. Nakashima K, Tran LS, Van Nguyen D, Fujita M, Maruyama K, Todaka D, Ito Y, Hayashi N, Shinozaki K, Yamaguchi-Shinozaki K: **Functional analysis of a NAC-type transcription factor OsNAC6 involved in abiotic and biotic stress-responsive gene expression in rice**. *The Plant journal : for cell and molecular biology* 2007, **51**(4):617-630.

19. Xiang Y, Huang Y, Xiong L: **Characterization of Stress-Responsive CIPK Genes in Rice for Stress Tolerance Improvement**. *Plant Physiology* 2007, **144**(3):1416-1428.

20. Saeng-ngam S, Takpirom W, Buaboocha T, Chadchawan S: **The role of the OsCam1-1 salt stress sensor in ABA accumulation and salt tolerance in rice**. *J Plant Biol* 2012, **55**(3):198-208.

21. Chinpongpanich A, Limruengroj K, Phean OPS, Limpaseni T, Buaboocha T: **Expression analysis of calmodulin and calmodulin-like genes from rice, Oryza sativa L**. *BMC Res Notes* 2012, **5**:625.

22. Dai X, Xu Y, Ma Q, Xu W, Wang T, Xue Y, Chong K: **Overexpression of an R1R2R3 MYB Gene, OsMYB3R-2, Increases Tolerance to Freezing, Drought, and Salt Stress in Transgenic Arabidopsis**. *Plant Physiology* 2007, **143**(4):1739-1751.

23. Yang A, Dai X, Zhang WH: **A R2R3-type MYB gene, OsMYB2, is involved in salt, cold, and dehydration tolerance in rice**. *J Exp Bot* 2012, **63**(7):2541-2556.

24. Asano T, Hakata M, Nakamura H, Aoki N, Komatsu S, Ichikawa H, Hirochika H, Ohsugi R: **Functional characterisation of OsCPK21, a calcium-dependent protein kinase that confers salt tolerance in rice**. *Plant molecular biology* 2011, **75**(1-2):179-191.

25. Cao Y, Wu Y, Zheng Z, Song F: **Overexpression of the rice EREBP-like gene OsBIERF3 enhances disease resistance and salt tolerance in transgenic tobacco**. *Physiological and Molecular Plant Pathology* 2005, **67**(3–5):202-211.

26. Liu D, Chen X, Liu J, Ye J, Guo Z: **The rice ERF transcription factor OsERF922 negatively regulates resistance to Magnaporthe oryzae and salt tolerance**. *J Exp Bot* 2012, **63**(10):3899-3911.

27. Liu AL, Zou J, Liu CF, Zhou XY, Zhang XW, Luo GY, Chen XB: **Over-expression of OsHsfA7 enhanced salt and drought tolerance in transgenic rice**. *BMB reports* 2013, **46**(1):31-36.

28. Wang Q, Guan Y, Wu Y, Chen H, Chen F, Chu C: **Overexpression of a rice OsDREB1F gene increases salt, drought, and low temperature tolerance in both Arabidopsis and rice**. *Plant molecular biology* 2008, **67**(6):589-602.

29. Fuchs I, Stolzle S, Ivashikina N, Hedrich R: **Rice K+ uptake channel OsAKT1 is sensitive to salt stress**. *Planta* 2005, **221**(2):212-221.

30. Li F, Guo S, Zhao Y, Chen D, Chong K, Xu Y: **Overexpression of a homopeptide repeat-containing bHLH protein gene (OrbHLH001) from Dongxiang Wild Rice confers freezing and salt tolerance in transgenic Arabidopsis**. *Plant cell reports* 2010, **29**(9):977-986.

31. Zhou J, Li F, Wang JL, Ma Y, Chong K, Xu YY: **Basic helix-loop-helix transcription factor from wild rice (OrbHLH2) improves tolerance to salt- and osmotic stress in Arabidopsis**. *Journal of plant physiology* 2009, **166**(12):1296-1306.

32. Choe YH, Kim YS, Kim IS, Bae MJ, Lee EJ, Kim YH, Park HM, Yoon HS: **Homologous expression of gamma-glutamylcysteine synthetase increases grain yield and tolerance of transgenic rice plants to environmental stresses**. *Journal of plant physiology* 2013, **170**(6):610-618.

33. Zhang JS, Xie C, Li ZY, Chen SY: **Expression of the plasma membrane H+-ATPase gene in response to salt stress in a rice salt-tolerant mutant and its original variety**. *Theoret Appl Genetics* 1999, **99**(6):1006-1011.

34. Singh AK, Kumar R, Pareek A, Sopory SK, Singla-Pareek SL: **Overexpression of rice CBS domain containing protein improves salinity, oxidative, and heavy metal tolerance in transgenic tobacco**. *Molecular biotechnology* 2012, **52**(3):205-216.

35. Sripinyowanich S, Klomsakul P, Boonburapong B, Bangyeekhun T, Asami T, Gu H, Buaboocha T, Chadchawan S: **Exogenous ABA induces salt tolerance in indica rice (Oryza sativa L.): The role of OsP5CS1 and OsP5CR gene expression during salt stress**. *Environmental and Experimental Botany* 2013, **86**(0):94-105.

36. Kanneganti V, Gupta AK: **Overexpression of OsiSAP8, a member of stress associated protein (SAP) gene family of rice confers tolerance to salt, drought and cold stress in transgenic tobacco and rice**. *Plant molecular biology* 2008, **66**(5):445-462.

37. Schmidt R, Schippers JH, Welker A, Mieulet D, Guiderdoni E, Mueller-Roeber B: **Transcription factor OsHsfC1b regulates salt tolerance and development in Oryza sativa ssp. japonica**. *AoB plants* 2012, **2012**:pls011.

38. Schmidt R, Mieulet D, Hubberten H-M, Obata T, Hoefgen R, Fernie AR, Fisahn J, San Segundo B, Guiderdoni E, Schippers JH: **SALT-RESPONSIVE ERF1 regulates reactive oxygen species–dependent signaling during the initial response to salt stress in rice**. *The Plant Cell Online* 2013, **25**(6):2115-2131.

39. Moons A, Gielen J, Vandekerckhove J, Straeten DVD, Gheysen G, Montagu MV: **An abscisic-acid- and salt-stress-responsive rice cDNA from a novel plant gene family**. *Planta* 1997, **202**(4):443-454.

40. Cao H, Guo S, Xu Y, Jiang K, Jones AM, Chong K: **Reduced expression of a gene encoding a Golgi localized monosaccharide transporter (OsGMST1) confers hypersensitivity to salt in rice (Oryza sativa)**. *J Exp Bot* 2011, **62**(13):4595-4604.

41. Wan B, Lin Y, Mou T: **Expression of rice Ca(2+)-dependent protein kinases (CDPKs) genes under different environmental stresses**. *FEBS Lett* 2007, **581**(6):1179-1189.

42. Ohtsu K, Ito Y, Saika H, NAKAZONO M, TSUTSUMI N, HIRAI A: **ABA-Independent Expression of Rice Alternative Oxidase Genes under Environmental Stresses**. *Plant Biotechnology* 2002, **19**(3):187-190.

43. Senadheera P, Singh RK, Maathuis FJ: **Differentially expressed membrane transporters in rice roots may contribute to cultivar dependent salt tolerance**. *J Exp Bot* 2009, **60**(9):2553-2563.

44. Hasthanasombut S, Supaibulwatana K, Mii M, Nakamura I: **Genetic manipulation of Japonica rice using the OsBADH1 gene from Indica rice to improve salinity tolerance**. *Plant Cell Tiss Organ Cult* 2011, **104**(1):79-89.

45. Jamil M, Iqbal W, Bangash A, Rehman SU, Imran QM, Rha ES: **Constitutive expression of OSC3H33, OSC3H50 and OSC3H37 genes in rice under salt stress**. *Pak J Bot* 2010, **42**:4003-4009.

46. Rus AM, Bressan RA, Hasegawa PM: **Unraveling salt tolerance in crops**. *Nature genetics* 2005, **37**(10):1029-1030.

47. Kumar G, Kushwaha H, Panjabi-Sabharwal V, Kumari S, Joshi R, Karan R, Mittal S, Pareek S, Pareek A: **Clustered metallothionein genes are co-regulated in rice and ectopic expression of OsMT1e-P confers multiple abiotic stress tolerance in tobacco via ROS scavenging**. *BMC Plant Biology* 2012, **12**(1):1-16.

48. Ye H, Du H, Tang N, Li X, Xiong L: **Identification and expression profiling analysis of TIFY family genes involved in stress and phytohormone responses in rice**. *Plant molecular biology* 2009, **71**(3):291-305.

49. Qiu D, Xiao J, Ding X, Xiong M, Cai M, Cao Y, Li X, Xu C, Wang S: **OsWRKY13 mediates rice disease resistance by regulating defense-related genes in salicylate- and jasmonate-dependent signaling**. *Molecular plant-microbe interactions : MPMI* 2007, **20**(5):492-499.

50. Chinpongpanich A, Limruengroj K, Phean-o-pas S, Limpaseni T, Buaboocha T: **Expression analysis of calmodulin and calmodulin-like genes from rice, Oryza sativa L**. *BMC Research Notes* 2012, **5**:625-625.

51. Xu GY, Rocha PS, Wang ML, Xu ML, Cui YC, Li LY, Zhu YX, Xia X: **A novel rice calmodulin-like gene, OsMSR2, enhances drought and salt tolerance and increases ABA sensitivity in Arabidopsis**. *Planta* 2011, **234**(1):47-59.

52. Macovei A, Vaid N, Tula S, Tuteja N: **A new DEAD-box helicase ATP-binding protein (OsABP) from rice is responsive to abiotic stress**. *Plant Signal Behav* 2012, **7**(9):1138-1143.

53. Hou X, Xie K, Yao J, Qi Z, Xiong L: **A homolog of human ski-interacting protein in rice positively regulates cell viability and stress tolerance**. *Proc Natl Acad Sci U S A* 2009, **106**(15):6410-6415.

54. Duan J, Zhang M, Zhang H, Xiong H, Liu P, Ali J, Li J, Li Z: **OsMIOX, a myo-inositol oxygenase gene, improves drought tolerance through scavenging of reactive oxygen species in rice (Oryza sativa L.)**. *Plant science : an international journal of experimental plant biology* 2012, **196**:143-151.

55. Yu S, Zhang X, Guan Q, Takano T, Liu S: **Expression of a carbonic anhydrase gene is induced by environmental stresses in rice (Oryza sativa L.)**. *Biotechnology letters* 2007, **29**(1):89-94.

56. Lu Z, Liu D, Liu S: **Two rice cytosolic ascorbate peroxidases differentially improve salt tolerance in transgenic Arabidopsis**. *Plant cell reports* 2007, **26**(10):1909-1917.

57. Park JJ, Yi J, Yoon J, Cho LH, Ping J, Jeong HJ, Cho SK, Kim WT, An G: **OsPUB15, an E3 ubiquitin ligase, functions to reduce cellular oxidative stress during seedling establishment**. *The Plant journal : for cell and molecular biology* 2011, **65**(2):194-205.

58. Luo M, Gu SH, Zhao SH, Zhang F, Wu NH: **Rice GTPase OsRacB: potential accessory factor in plant salt-stress signaling**. *Acta biochimica et biophysica Sinica* 2006, **38**(6):393-402.

59. Gao T, Wu Y, Zhang Y, Liu L, Ning Y, Wang D, Tong H, Chen S, Chu C, Xie Q: **OsSDIR1 overexpression greatly improves drought tolerance in transgenic rice**. *Plant molecular biology* 2011, **76**(1-2):145-156.

60. Liu HL, Dai XY, Xu YY, Chong K: **Over-expression of OsUGE-1 altered raffinose level and tolerance to abiotic stress but not morphology in Arabidopsis**. *Journal of plant physiology* 2007, **164**(10):1384-1390.

61. Kumar K, Rao KP, Biswas DK, Sinha AK: **Rice WNK1 is regulated by abiotic stress and involved in internal circadian rhythm**. *Plant Signal Behav* 2011, **6**(3):316-320.

62. Liu D, Zhang X, Cheng Y, Takano T, Liu S: **rHsp90 gene expression in response to several environmental stresses in rice (Oryza sativa L.)**. *Plant physiology and biochemistry : PPB / Societe francaise de physiologie vegetale* 2006, **44**(5-6):380-386.

63. Wu TM, Lin WR, Kao YT, Hsu YT, Yeh CH, Hong CY, Kao CH: **Identification and characterization of a novel chloroplast/mitochondria co-localized glutathione reductase 3 involved in salt stress response in rice**. *Plant molecular biology* 2013, **83**(4-5):379-390.

64. Wang S, Uddin MI, Tanaka K, Yin L, Shi Z, Qi Y, Mano J, Matsui K, Shimomura N, Sakaki T *et al*: **Maintenance of Chloroplast Structure and Function by Overexpression of the Rice MONOGALACTOSYLDIACYLGLYCEROL SYNTHASE Gene Leads to Enhanced Salt Tolerance in Tobacco**. *Plant Physiol* 2014, **165**(3):1144-1155.

65. Agrawal GK, Agrawal SK, Shibato J, Iwahashi H, Rakwal R: **Novel rice MAP kinases OsMSRMK3 and OsWJUMK1 involved in encountering diverse environmental stresses and developmental regulation**. *Biochemical and biophysical research communications* 2003, **300**(3):775-783.

66. Jain M, Tyagi AK, Khurana JP: **Overexpression of putative topoisomerase 6 genes from rice confers stress tolerance in transgenic Arabidopsis plants**. *The FEBS journal* 2006, **273**(23):5245-5260.

67. Huang YW, Tsay WS, Chen CC, Lin CW, Huang HJ: **Increased expression of the rice C-type cyclin-dependent protein kinase gene, Orysa;CDKC;1, in response to salt stress**. *Plant physiology and biochemistry : PPB / Societe francaise de physiologie vegetale* 2008, **46**(1):71-81.

68. Nakamura A, Fukuda A, Sakai S, Tanaka Y: **Molecular cloning, functional expression and subcellular localization of two putative vacuolar voltage-gated chloride channels in rice (Oryza sativa L.)**. *Plant & cell physiology* 2006, **47**(1):32-42.

69. Qi Y, Kawano N, Yamauchi Y, Ling J, Li D, Tanaka K: **Identification and cloning of a submergence-induced gene OsGGT (glycogenin glucosyltransferase) from rice (Oryza sativa L.) by suppression subtractive hybridization**. *Planta* 2005, **221**(3):437-445.

70. Qin Y, Ye H, Tang N, Xiong L: **Systematic identification of X1-homologous genes reveals a family involved in stress responses in rice**. *Plant molecular biology* 2009, **71**(4-5):483-496.

71. Liu C, Mao B, Ou S, Wang W, Liu L, Wu Y, Chu C, Wang X: **OsbZIP71, a bZIP transcription factor, confers salinity and drought tolerance in rice**. *Plant molecular biology* 2014, **84**(1-2):19-36.

72. Jeong M-J, Lee S-K, Kim B-G, Kwon T-R, Cho W-S, Park Y-T, Lee J-O, Kwon H-B, Byun M-O, Park S-C: **A rice (Oryza sativa L.) MAP kinase gene, OsMAPK44, is involved in response to abiotic stresses**. *Plant Cell, Tissue and Organ Culture* 2006, **85**(2):151-160.

73. Ning J, Li X, Hicks LM, Xiong L: **A Raf-like MAPKKK gene DSM1 mediates drought resistance through reactive oxygen species scavenging in rice**. *Plant physiology* 2010, **152**(2):876-890.

74. Guan Q, Xia D, Liu S: **OsAPX4 gene response to several environmental stresses in rice (Oryza sativa L.)**. *African Journal of Biotechnology* 2013, **9**(36).

75. Wani SH, Gosal SS: **Introduction of OsglyII gene into Oryza sativa for increasing salinity tolerance**. *Biologia Plantarum* 2011, **55**(3):536-540.

76. Guo X, Wu Y, Wang Y, Chen Y, Chu C: **OsMSRA4. 1 and OsMSRB1. 1, two rice plastidial methionine sulfoxide reductases, are involved in abiotic stress responses**. *Planta* 2009, **230**(1):227-238.

77. Vannini C, Locatelli F, Bracale M, Magnani E, Marsoni M, Osnato M, Mattana M, Baldoni E, Coraggio I: **Overexpression of the rice Osmyb4 gene increases chilling and freezing tolerance of Arabidopsis thaliana plants**. *The Plant journal : for cell and molecular biology* 2004, **37**(1):115-127.

78. Qiao W, Xiao S, Yu L, Fan L-M: **Expression of a rice gene OsNOA1 re-establishes nitric oxide synthesis and stress-related gene expression for salt tolerance in Arabidopsis nitric oxide-associated 1 mutant Atnoa1**. *Environmental and Experimental Botany* 2009, **65**(1):90-98.

79. Nayidu NK, Wang L, Xie W, Zhang C, Fan C, Lian X, Zhang Q, Xiong L: **Comprehensive sequence and expression profile analysis of PEX11 gene family in rice**. *Gene* 2008, **412**(1-2):59-70.

80. Huang J, Wang M-M, Bao Y-M, Sun S-J, Pan L-J, Zhang H-S: **SRWD: A novel WD40 protein subfamily regulated by salt stress in rice (OryzasativaL.)**. *Gene* 2008, **424**(1–2):71-79.

81. Huang J, Wang M-M, Jiang Y, Wang Q-H, Huang X, Zhang H-S: **Stress repressive expression of rice SRZ1 and characterization of plant SRZ gene family**. *Plant Science* 2008, **174**(2):227-235.

82. Macovei A, Tuteja N: **microRNAs targeting DEAD-box helicases are involved in salinity stress response in rice (Oryza sativa L.)**. *BMC Plant Biol* 2012, **12**:183.

83. Roy M, Wu R: **Overexpression of S-adenosylmethionine decarboxylase gene in rice increases polyamine level and enhances sodium chloride-stress tolerance**. *Plant Science* 2002, **163**(5):987-992.

84. Kim SK, You YN, Park JC, Joung Y, Kim BG, Ahn JC, Cho HS: **The rice thylakoid lumenal cyclophilin OsCYP20-2 confers enhanced environmental stress tolerance in tobacco and Arabidopsis**. *Plant cell reports* 2012, **31**(2):417-426.

85. Jin X, Xue Y, Wang R, Xu R, Bian L, Zhu B, Han H, Peng R, Yao Q: **Transcription factor OsAP21 gene increases salt/drought tolerance in transgenic Arabidopsis thaliana**. *Molecular biology reports* 2013, **40**(2):1743-1752.

86. Teixeira FK, Menezes-Benavente L, Galvão VC, Margis R, Margis-Pinheiro M: **Rice ascorbate peroxidase gene family encodes functionally diverse isoforms localized in different subcellular compartments**. *Planta* 2006, **224**(2):300-314.

87. Huda KM, Banu MS, Garg B, Tula S, Tuteja R, Tuteja N: **OsACA6, a P-type IIB Ca(2)(+) ATPase promotes salinity and drought stress tolerance in tobacco by ROS scavenging and enhancing the expression of stress-responsive genes**. *The Plant journal : for cell and molecular biology* 2013, **76**(6):997-1015.

88. Alam MM, Tanaka T, Nakamura H, Ichikawa H, Kobayashi K, Yaeno T, Yamaoka N, Shimomoto K, Takayama K, Nishina H *et al*: **Overexpression of a rice heme activator protein gene (OsHAP2E) confers resistance to pathogens, salinity and drought, and increases photosynthesis and tiller number**. *Plant biotechnology journal* 2015, **13**(1):85-96.

89. Kader MA, Seidel T, Golldack D, Lindberg S: **Expressions of OsHKT1, OsHKT2, and OsVHA are differentially regulated under NaCl stress in salt-sensitive and salt-tolerant rice (Oryza sativa L.) cultivars**. *J Exp Bot* 2006, **57**(15):4257-4268.

90. Lakra N, Nutan KK, Das P, Anwar K, Singla-Pareek SL, Pareek A: **A nuclear-localized histone-gene binding protein from rice (OsHBP1b) functions in salinity and drought stress tolerance by maintaining chlorophyll content and improving the antioxidant machinery**. *Journal of plant physiology* 2014, **176c**:36-46.

91. Kusuda H, Koga W, Kusano M, Oikawa A, Saito K, Hirai MY, Yoshida KT: **Ectopic expression of myo-inositol 3-phosphate synthase induces a wide range of metabolic changes and confers salt tolerance in rice**. *Plant science : an international journal of experimental plant biology* 2015, **232**:49-56.

92. Xiong H, Li J, Liu P, Duan J, Zhao Y, Guo X, Li Y, Zhang H, Ali J, Li Z: **Overexpression of OsMYB48-1, a novel MYB-related transcription factor, enhances drought and salinity tolerance in rice**. *PLoS One* 2014, **9**(3):e92913.

93. Matsuda S, Nagasawa H, Yamashiro N, Yasuno N, Watanabe T, Kitazawa H, Takano S, Tokuji Y, Tani M, Takamure I *et al*: **Rice RCN1/OsABCG5 mutation alters accumulation of essential and nonessential minerals and causes a high Na/K ratio, resulting in a salt-sensitive phenotype**. *Plant science : an international journal of experimental plant biology* 2014, **224**:103-111.

94. Wu H, Ye H, Yao R, Zhang T, Xiong L: **OsJAZ9 acts as a transcriptional regulator in jasmonate signaling and modulates salt stress tolerance in rice**. *Plant science : an international journal of experimental plant biology* 2015, **232**:1-12.

95. Jin S, Sun D, Wang J, Li Y, Wang X, Liu S: **Expression of the rgMT gene, encoding for a rice metallothionein-like protein in Saccharomyces cerevisiae and Arabidopsis thaliana**. *Journal of genetics* 2014, **93**(3):709-718.

96. Tan CM, Chen RJ, Zhang JH, Gao XL, Li LH, Wang PR, Deng XJ, Xu ZJ: **OsPOP5, a prolyl oligopeptidase family gene from rice confers abiotic stress tolerance in Escherichia coli**. *International journal of molecular sciences* 2013, **14**(10):20204-20219.
