# Supplementary figures and images for: Identification and analysis of novel salt responsive candidate gene based SSRs (cgSSRs) from rice (Oryza sativa L.)
Source: BMC Plant Biol. 2015 May 16;15:122. doi: 10.1186/s12870-015-0498-1 (PMC4435636; doi:10.1186/s12870-015-0498-1)

## Slide 1
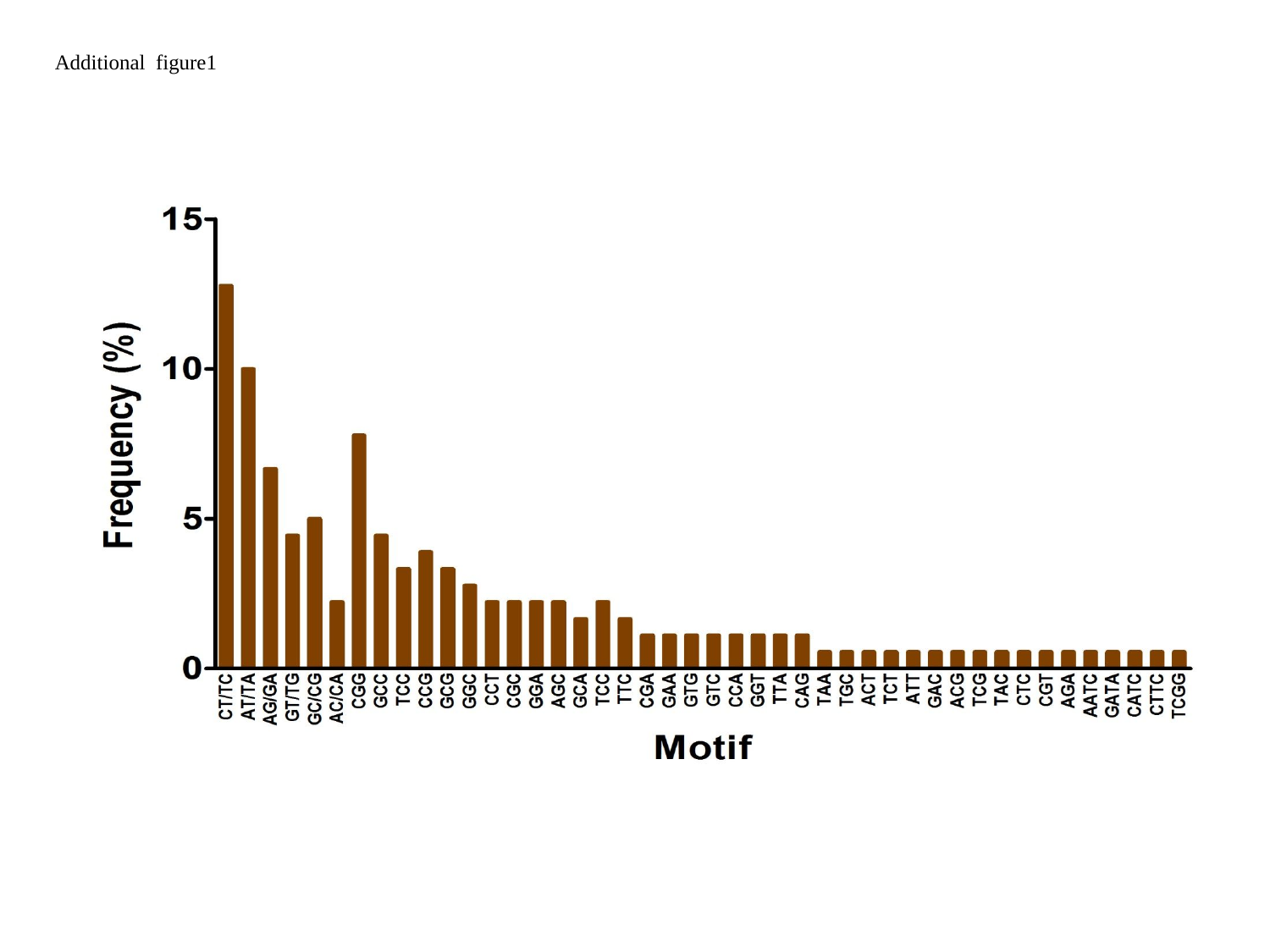

Additional figure1

Supplement: Additional file 3: — Bar diagram showing frequency of different types of cgSSR motifs found in salt responsive candidate genes of rice. [file 12870_2015_498_MOESM3_ESM.pptx]
